# Predictors of Mortality among Patients Hospitalized with COVID-19 during the First Wave in India: A Multisite Case-Control Study

**DOI:** 10.4269/ajtmh.22-0705

**Published:** 2023-03-13

**Authors:** Anand Krishnan, Rakesh Kumar, Ritvik Amarchand, Anant Mohan, Ravi Kant, Ankit Agarwal, Poorvi Kulshreshtha, Prasan Kumar Panda, Ajeet Singh Bhadoria, Neeraj Agarwal, Bijit Biswas, Rathish Nair, Naveet Wig, Rajesh Malhotra, Sushma Bhatnagar, Richa Aggarwal, Kapil Dev Soni, Nirupam Madan, Anjan Trikha, Pawan Tiwari, Angel Rajan Singh, Mukta Wyawahare, Venugopalan Gunasekaran, Dineshbabu Sekar, Sanjeev Misra, Pankaj Bhardwaj, Akhil Dhanesh Goel, Naveen Dutt, Deepak Kumar, Nitin M. Nagarkar, Abhiruchi Galhotra, Atul Jindal, Utsav Raj, Ajoy Behera, Sabbah Siddiqui, Arun Kokane, Rajnish Joshi, Abhijit Pakhare, Farhan Farooque, Sai Pawan, Pradeep Deshmukh, Ranjan Solanki, Bharatsing Rathod, Vibha Dutta, Prasanta Raghab Mohapatra, Manoj Kumar Panigrahi, Sadananda Barik, Randeep Guleria

**Affiliations:** ^1^ Centre for Community Medicine, All India Institute of Medical Sciences (AIIMS), New Delhi, India;; ^2^ Department of Pulmonary, Critical Care and Sleep Medicine, AIIMS, New Delhi, India;; ^3^ AIIMS, Rishikesh, India;; ^4^ Department of Anaesthesia, AIIMS, Rishikesh, India;; ^5^ Department of Physiology, AIIMS, Rishikesh, India;; ^6^ Department of Medicine, AIIMS, Rishikesh, India;; ^7^ Department of Community & Family Medicine, AIIMS, Rishikesh, India;; ^8^ Department of Community & Family Medicine, AIIMS, Patna, India;; ^9^ College of Nursing, AIIMS, Patna, India;; ^10^ Department of Medicine, AIIMS, New Delhi, India;; ^11^ Jai Prakash Narayan Apex Trauma Center, AIIMS, New Delhi, India;; ^12^ Department of Onco-Anaesthesia, BRAIRCH, AIIMS, New Delhi, India;; ^13^ Department of Anaesthesia and Critical Care, Jai Prakash Narayan Apex Trauma Center, AIIMS, New Delhi, India;; ^14^ Department of Hospital Administration, AIIMS, New Delhi, India;; ^15^ Department of Anaesthesiology, Pain Medicine and Critical Care, AIIMS, New Delhi, India;; ^16^ Department of Medicine, Jawaharlal Institute of Postgraduate Medical Education & Research (JIPMER), India;; ^17^ Department of Geriatric Medicine, JIPMER, India;; ^18^ AIIMS, Jodhpur, India;; ^19^ Department of Community & Family Medicine, AIIMS, Jodhpur, India;; ^20^ Department of Pulmonary Medicine, AIIMS, Jodhpur, India;; ^21^ Department of Medicine, AIIMS, Jodhpur, India;; ^22^ AIIMS, Raipur, India;; ^23^ Department of Community & Family Medicine, AIIMS, Raipur, India;; ^24^ Department of Paediatrics, AIIMS, Raipur, India;; ^25^ National Tuberculosis Elimination Program, AIIMS, Raipur, India;; ^26^ Department of Pulmonary Medicine, AIIMS, Raipur, India;; ^27^ Department of Medicine, AIIMS, Raipur, India;; ^28^ Department of Community & Family Medicine, AIIMS, Bhopal, India;; ^29^ Department of Medicine, AIIMS, Bhopal, India;; ^30^ Department of Community & Family Medicine, AIIMS, Nagpur, India;; ^31^ Department of Medicine, AIIMS, Nagpur, India;; ^32^ AIIMS, Nagpur, India;; ^33^ Department of Pulmonary Medicine & Critical Care, AIIMS, Bhubaneswar, India;; ^34^ Department of Trauma & Emergency Medicine, AIIMS, Bhubaneswar, India;; ^35^ AIIMS, New Delhi, India

## Abstract

Severe acute respiratory syndrome coronavirus 2 disease (COVID-19) has caused more than 6 million deaths globally. Understanding predictors of mortality will help in prioritizing patient care and preventive approaches. This was a multicentric, unmatched, hospital-based case-control study conducted in nine teaching hospitals in India. *Cases were microbiologically confirmed COVID-19 patients who died in the hospital during the period of study and controls were microbiologically confirmed COVID-19 patients who were discharged from the same hospital after recovery*. Cases were recruited sequentially from March 2020 until December–March 2021. All information regarding cases and controls was extracted retrospectively from the medical records of patients by trained physicians. Univariable and multivariable logistic regression was done to assess the association between various predictor variables and deaths due to COVID-19. A total of 2,431 patients (1,137 cases and 1,294 controls) were included in the study. The mean age of patients was 52.8 years (SD: 16.5 years), and 32.1% were females. Breathlessness was the most common symptom at the time of admission (53.2%). Increasing age (adjusted odds ratio [aOR]: 46–59 years, 3.4 [95% CI: 1.5–7.7]; 60–74 years, 4.1 [95% CI: 1.7–9.5]; and ≥ 75 years, 11.0 [95% CI: 4.0–30.6]); preexisting diabetes mellitus (aOR: 1.9 [95% CI: 1.2–2.9]); malignancy (aOR: 3.1 [95% CI: 1.3–7.8]); pulmonary tuberculosis (aOR: 3.3 [95% CI: 1.2–8.8]); breathlessness at the time of admission (aOR: 2.2 [95% CI: 1.4–3.5]); high quick Sequential Organ Failure Assessment score at the time of admission (aOR: 5.6 [95% CI: 2.7–11.4]); and oxygen saturation < 94% at the time of admission (aOR: 2.5 [95% CI: 1.6–3.9]) were associated with mortality due to COVID-19. These results can be used to prioritize patients who are at increased risk of death and to rationalize therapy to reduce mortality due to COVID-19.

## INTRODUCTION

The pandemic of novel severe acute respiratory syndrome coronavirus 2 (SARS-CoV-2), first reported in the Wuhan province in China in December 2019, caused at least 3 million deaths globally in 2020 by the WHO’s preliminary estimates.^1^ With vaccination gaining pace across the globe, there has been a decline in mortality due to SARS-CoV-2 disease (COVID-19); however, COVID-19 is still causing substantial mortality.^2^ In addition, the likelihood of the emergence of new variants, leading to a surge in cases and deaths, remains a definite possibility. In-hospital case fatality due to COVID-19 has been variously reported as 2–3%.^3^

The mortality observed in the Indian and South Asian subcontinent has reportedly been lower than in the West, and multiple reasons have been ascribed for this differential mortality as described in the literature.^4^ Understanding of the predictors of mortality due to COVID-19 helps in prioritizing patient care, especially in low-resource settings where hospital beds are limited. It also helps in prioritizing preventive care and vaccination. An understanding of the predisposing conditions and disease-specific clinical, laboratory, and radiological parameters can assist in developing a COVID-19–specific composite score to predict unfavorable clinical outcomes.^5^

Various studies have reported higher age, male sex, low oxygen saturation (SpO_2_) and dyspnea at admission, and preexisting morbidities as predictors of in-hospital mortality due to COVID-19.^6^^–^^8^ However, data on the effect of various repurposed and other therapies are contradictory. Few studies reported beneficial effect of drugs like hydroxychloroquine in preventing death;^9^ most randomized controlled trials did not corroborate this.^10^^,^^11^ There are limited data from India on either the predictors of COVID-19 deaths or the effect of various drugs and treatment modalities in preventing death, and the available data are from a single health facility, restricting their generalizability.^12^^,^^13^ This multicentric hospital-based study was undertaken with the objective of documenting treatment practices and identifying predictors of mortality among symptomatic COVID-19 patients who were admitted to different hospitals.

## MATERIALS AND METHODS

This retrospective, hospital-based, unmatched case-control study was assessed among deaths that occurred at nine tertiary care teaching hospitals in India between March 2020 through December 2020 to March 2021. Study hospitals were conveniently chosen to represent different regions of the country. Their capacity varied from 60 to 2,700 beds, and all were managing COVID-19 as well as non–COVID-19 cases.

### Case and control selection.

Cases were defined as patients with SARS CoV-2 infection confirmed by real-time reverse transcriptase polymerase chain reaction (RT-PCR)/rapid antigen test (RAT) during the hospital stay or within 28 days before hospital admission and death in the hospital during the study period. Controls were patients with infection with SARS CoV-2 diagnosed by RT-PCR/RAT who were discharged from the hospital after recovery and were recruited from the same hospital. Although we initially planned to have two controls per case, this was later changed to one control per case when the sample size planned for the study (660 cases) was exceeded. This sample size was adequate for estimation of an odds ratio of 1.5 for a population exposure level of 15% with 1% alpha error and 85% power. Cases were consecutively selected from hospital records by date of admission. Cases and controls were selected at a ratio of 1:1. Controls were not matched to cases except by day of admission in the same hospital and were selected randomly from hospital records.

### Data extraction.

All information regarding cases and controls was extracted retrospectively from the medical records of patients. A standardized data extraction sheet was developed, and data were extracted by trained physicians. In addition to age and sex, information on preexisting morbidities (i.e., hypertension, diabetes mellitus, ischemic heart disease, stroke, any malignancy, chronic respiratory disease, tuberculosis, and immune disorder, as well as tobacco smoking) was extracted. Missing information on preexisting morbidities was treated as absence of morbidity, as it was assumed that it is a routine practice to record those morbidities in hospital case sheets. Information on the clinical status of a patient at the time of admission, that is, presenting symptoms (cough, fever, difficulty in breathing) and signs (respiratory rate, systolic and diastolic blood pressure, altered mental status, SpO_2_, and pulse rate), was extracted. The quick Sequential Organ Failure Assessment (qSOFA) score was calculated by assigning a score of 1 each for altered mental status, respiratory rate ≥ 22, and systolic blood pressure ≥ 100 mm Hg. Information on administration of antivirals, steroids, hydroxychloroquine, convalescent plasma, or immunoglobulins was collected; however, data on type of antiviral or duration of drug treatment were not extracted. In addition, information on type of respiratory support (high-flow nasal cannulation, noninvasive and invasive mechanical ventilation), complications during the hospital stay (sepsis, heart failure, respiratory failure, coagulopathy, acute cardiac and kidney injury, stroke, arrythmia, and myocarditis), and admission to the intensive care unit (ICU) was extracted.

### Data analysis.

Data were entered in an Excel sheet and imported in Stata version 15 (StataCorp, College Station, TX) for further analysis. No imputation was done for missing data. Various study characteristics were presented either as proportion or means and compared between cases and controls using the χ^2^ test or unpaired *t*-test wherever appropriate. Univariable and multivariable logistic regression was done to assess the association between various predictor variables and deaths due to COVID-19. To assess the association between demographic variables and clinical presentation, the multivariable logistic regression was adjusted for variables that were found to be significant (*P* < 0.05) in the bivariate analysis. Adjustments were done in the multivariable logistic regression for age, sex, preexisting morbidities, presenting symptoms at the time of admission, qSOFA score and SpO_2_ at the time of admission, admission in the ICU, invasive mechanical ventilation, and associated complications in the hospital. Adjustment for clustering by site was done in all analyses. *P* < 0.05 was considered significant. Ethical approval for the analysis was obtained from the Ethics Committee of the All India Institute of Medical Sciences, New Delhi, and all participating institutes.

## RESULTS

A total of 2,431 patients were included in the study; 1,137 were cases, and 1,294 were controls. The least number of cases contributed by a site was 27, and the maximum was 334. The participating hospitals showed considerable variations in patient severity profile (comorbidity and qSOFA score) and treatment practices, especially the use of antivirals and hydroxychloroquine (Table 1).

**Table 1:** Profile of participating hospitals based on the study sample

Hospital	Study period	Total cases	Total controls	% Of controls with		Treatment practices among controls (%)				Outcomes in the hospital among controls (%)	
				Co- morbidity	qSOFA score > 1	Antibiotic use	Anti-viral use	Steroid use	Hydroxy- chloroquine use	Invasive mechanical ventilation	Intensive care unit admission
1	Mar 20–Dec 20	52	86	45.4	2.1	47.7	3.5	19.8	3.5	0	0
2	July 20–Sep 20	27	27	29.6	0	37.0	44.4	22.2	18.5	0	0
3	Apr 20–Mar 21	130	138	22.5	0	45.7	6.5	10.9	0	2.9	10.9
4	June 20–Jan 21	32	58	44.8	0	82.8	69.0	44.8	15.5	0	0
5	May 20–July 20	219	173	37.6	2.3	99.4	5.2	48.0	93.6	0.6	2.3
6	June 20–Dec 20	62	120	49.2	5.8	77.5	3.3	32.5	59.2	0.8	13.3
7	Apr 20–Dec 20	334	324	30.9	2.2	36.7	2.5	12.4	0.9	11.8	7.1
8	May 20–Dec 20	117	208	42.3	2.7	24.0	6.7	21.6	0	0	3.8
9	Apr 20–Dec 20	164	160	62.5	6.4	94.4	5.6	47.5	67.5	3.8	40.0
Total	–	1,137	1,294	39.9	2.9	57.7	8.4	26.8	27.9	3.9	10.0

qSOFA = quick Sequential Organ Failure Assessment.

The mean age of patients was 52.8 years (SD: 16.5 years), and 32.1% were females. In addition, 6.1% of patients had a history of ischemic heart disease, one-third had a history of hypertension, approximately one-third had a history of diabetes mellitus, 10.0% had a history of chronic respiratory disease, and 12.2% were current smokers (Table 2). Only half were diagnosed as COVID-19 cases before admission to the hospital; the rest were diagnosed after admission to the hospital. Breathlessness was the most common symptom at the time of admission (53.1%). An antibiotic was prescribed for 73.7% of patients, whereas antiviral medications were prescribed for only 16.2% of patients. Hydroxychloroquine was prescribed for 24.8% of patients, and steroids were prescribed for 47.2% of patients. In addition, 38.6% of patients were put on mechanical ventilation, and 41.9% were admitted to the ICU (Table 2).

**Table 2:** Demographic and clinical characteristics of patients who died as a result of COVID-19 and their unmatched controls in nine hospitals in India

Characteristics	Total (*N* = 2,431)	Cases (*N* = 1,137)	Controls (*N* = 1,294)	*P* value
Mean (SD) age (years)	52.8 (16.5)	59.3 (15.1)	47.1 (15.6)	< 0.001
Age group (years)				
18–30	303 (12.5)	58 (5.1)	245 (18.9)	< 0.001
31–45	533 (21.9)	155 (13.6)	378 (29.2)	–
46–59	651(26.8)	303 (26.7)	348 (26.9)	–
60–74	712 (29.3)	442 (38.9)	270 (20.9)	–
≥ 75	232 (9.5)	179 (15.7)	53 (4.1)	–
Sex				< 0.001
Male	1,650 (67.9)	812 (71.4)	838 (64.8)	–
Female	781 (32.1)	325 (28.6)	456 (35.2)	–
Preexisting morbidities or risk factors				
Coronary artery disease	149 (6.1)	98 (8.4)	53 (4.1)	< 0.001
Hypertension	821 (33.8)	524 (46.1)	297 (23.0)	< 0.001
Stroke	39 (1.6)	28 (2.5)	11 (0.9)	0.002
Diabetes mellitus	854 (35.1)	541 (47.6)	313 (24.2)	< 0.001
Any malignancy	76 (3.1)	46 (4.1)	30 (2.3)	0.015
Chronic respiratory disease	242 (10.0)	195 (17.1)	47 (3.6)	< 0.001
Pulmonary tuberculosis	74 (3.0)	45 (4.0)	29 (2.2)	0.014
Immune disorder	35 (1.4)	30 (2.6)	5 (0.4)	< 0.001
Smoking	296 (12.2)	167 (14.7)	129 (10.0)	< 0.001
Admitted as COVID-19 case	1,164 (47.9)	368 (32.4)	796 (61.5)	< 0.001
Presenting symptoms at admission				
Fever	1,137 (46.8)	610 (53.7)	527 (40.7)	< 0.001
Cough	1,094 (45.0)	574 (50.2)	525 (40.5)	< 0.001
Breathlessness	1,290 (53.1)	880 (77.5)	410 (31.7)	< 0.001
Altered mental status	187 (7.7)	167 (14.7)	20 (1.5)	< 0.001
Clinical status at admission				
Mean respiratory rate (SD)*	25.7 (14.9)	26.7 (11.0)	24.8 (17.6)	0.003
Mean Spo_2_ (SD)†	91.7 (11.3)	86.7 (13.9)	96.2 (4.9)	< 0.001
Mean SBP (SD)‡	128.2 (23.2)	129.8 (26.8)	126.8 (19.3)	0.002
qSOFA score§				
0	807 (36.8)	203 (19.5)	604 (52.3)	–
1	1,146 (52.2)	629 (60.5)	517 (44.8)	0.001
2	231 (10.5)	197 (18.9)	34 (2.9)	–
3	11 (0.5)	11 (1.1)	0	–
Medications during hospital stay				
Antivirals	394 (16.2)	286 (25.2)	109 (8.4)	< 0.001
Steroids	1,147 (47.2)	800 (70.4)	347 (26.8)	< 0.001
Hydroxychloroquine	603 (24.8)	242 (21.3)	361 (27.9)	< 0.001
Intravenous immunoglobulin	74 (3.0)	65 (5.7)	9 (0.7)	< 0.001
Convalescent plasma	99 (4.1)	94 (8.3)	5 (0.4)	< 0.001
Respiratory or other clinical support				
High-flow nasal cannulation	583 (24.0)	476 (41.9)	107 (8.3)	< 0.001
Noninvasive ventilation	558 (23.0)	541 (47.6)	17 (1.3)	< 0.001
Invasive ventilation	939 (38.6)	889 (78.2)	50 (3.9)	< 0.001
ECMO	5 (0.2)	4 (0.4)	1 (0.1)	0.136
Dialysis	102 (4.2)	84 (7.4)	18 (1.4)	< 0.001
Admitted to intensive care unit	1,018 (41.9)	888 (78.2)	130 (10.1)	< 0.001
Associated complications				
Sepsis	409 (16.8)	400 (35.2)	9 (0.7)	< 0.001
Coagulopathy	145 (6.0)	99 (8.7)	46 (3.6)	< 0.001
Acute renal injury	348 (14.3)	337 (29.6)	11 (0.9)	< 0.001
Stroke	40 (1.6)	40 (3.5)	0	< 0.001
Arrythmia	72 (3.0)	72 (6.3)	0	< 0.001
Myocarditis	23 (1.0)	23 (2.0)	0	< 0.001

ECMO = zxtracorporeal membrane oxygenation; qSOFA = quick Sequential Organ Failure Assessment; SBP = systolic blood pressure; Spo_2_ = oxygen saturation.

* *n* Case: 1,059; control: 1,179.

† *n* Case: 1,089; control: 1,217.

‡ *n* Case: 1,080, control: 1,220.

§ *n* Case: 1,040; control: 1,155.

On bivariate analysis, cases were more likely than controls to be older (59.3 years versus 47.1 years) and male (71.4% versus 64.8%) and to have comorbidities (ischemic heart disease: 8.4% versus 4.1%; hypertension: 46.1% versus 23.0%; stroke: 2.5% versus 0.9%; diabetes mellitus: 47.6% versus 24.2%; chronic respiratory disease: 17.1% versus 3.6%; and an immune disorder: 2.6% versus 0.4%). Cases were less likely than controls to have been admitted with confirmed COVID-19 (32.4% versus 61.5%) and more likely to have a fever (53.7% versus 40.7%), and breathlessness (77.5% versus 31.7%). Cases also had a lower mean SpO_2_ than controls (86.7% versus 96.2%) at the time of admission. Cases were more likely than controls to receive antivirals (25.2% versus 8.4%) and steroids (70.4% versus 26.8%) but less likely to receive hydroxychloroquine (21.3% versus 27.9%), more likely to be put on invasive (78.2% versus 3.9%) or noninvasive (47.6% versus 1.3%) ventilation, and more likely to be admitted to the ICU (78.2% versus 10.1%). During treatment, cases were more likely than controls to develop sepsis (35.2% versus 0.7%), coagulopathy (8.7% versus 3.6%), acute renal injury (29.6% versus 0.9%), stroke (3.5% versus 0), and myocarditis (2.0% versus 0) (Table 2).

On multivariable analysis, the following variables were associated with mortality due to COVID-19: increasing age (adjusted odds ratio [aOR]: 46–59 years: 3.4 [95% CI: 1.5–7.7]), 60–74 years: (4.1 [95% CI: 1.7–9.5]), ≥ 75 years: (11.0 [95% CI: 4.0–30.6]); preexisting diabetes mellitus (aOR: 1.9 [95% CI: 1.2–2.9]); malignancy (aOR: 3.1 [95% CI: 1.3–7.8]); pulmonary tuberculosis (aOR: 3.3 [95% CI: 1.2–8.8]); breathlessness at the time of admission (aOR: 2.2 [95% CI: 1.4–3.5]); high qSOFA score at the time of admission (aOR: 5.6 [95% CI: 2.7–11.4]); and SpO_2_ < 94% at the time of admission (aOR: 2.5 [95% CI: 1.6–3.9]).

There was no difference in mortality by sex after adjustment for other variables. Administration of convalescent plasma or hydroxychloroquine during hospitalization did not affect the odds of mortality (Table 3). People who were put on mechanical ventilation (aOR: 32.6 [95% CI: 19.6–54.2]) or admitted to the ICU (aOR: 8.4 [95% CI: 5.5–12.9]) had very strong odds of mortality due to COVID-19.

**Table 3:** Association between demographics, clinical characteristics, and mortality among COVID-19 patients in nine hospitals in India

Characteristic	Unadjusted OR (95% CI of OR)	*P* value	Adjusted OR* (95% CI of OR)	*P* value
Age group (years)				
≤ 30	Ref	–	(Ref)	–
31–45	1.7 (1.2–2.4)	0.002	1.7 (0.8–3.9)	0.197
46–59	3.6 (2.7–5.1)	< 0.001	3.8 (1.5–7.7)	0.004
60–74	6.9 (5.0–9.6)	< 0.001	4.1 (1.7–9.5)	0.001
≥ 75	14.3 (9.4–21.7)	< 0.001	11.0 (4.0–30.6)	< 0.001
Sex				
Female	Ref	–	Ref	–
Male	1.4 (1.1–1.6)	< 0.001	1.3 (0.9–2.0)	0.183
Preexisting morbidity				
Coronary artery disease	2.2 (1.5–3.1)	< 0.001	0.5 (0.1–1.2)	0.118
Hypertension	2.9 (2.4–3.4)	< 0.001	1.1 (0.7–1.7)	0.716
Stroke	2.9 (1.5–5.9)	0.003	0.7 (0.2–3.7)	0.709
Diabetes mellitus	2.8 (2.4–3.4)	< 0.001	1.9 (1.2–2.9)	0.004
Any malignancy	1.8 (1.1–2.8)	0.016	3.1 (1.3–7.8)	0.013
Chronic respiratory disease	5.5 (3.9–7.6)	< 0.001	0.8 (0.4–1.6)	0.523
Pulmonary tuberculosis	1.8 (1.1–2.9)	0.015	3.3 (1.2–8.7)	0.018
Immune disorder	6.9 (2.7–18.1)	< 0.001	1.9 (0.3–11.9)	0.478
Admitted as COVID-19 case	0.3 (0.2–0.4)	< 0.001	0.7 (0.5–1.0)	0.068
Presenting symptoms at admission				
Fever	1.7 (1.4–2.0)	< 0.001	1.4 (0.9–2.1)	0.093
Cough	1.5 (1.3–1.7)	< 0.001	0.8 (0.5–1.2)	0.274
Breathlessness	7.4 (6.2–9.0)	< 0.001	2.2 (1.4–3.5)	< 0.001
Clinical status at admission				
SpO_2_ (< 94%)	9.4 (7.6–11.5)	< 0.001	2.5 (1.6–3.9)	< 0.001
qSOFA				
Low (0–1)	Ref	–	Ref	–
High (2–3)	8.2 (5.7–12.0)	< 0.001	5.6 (2.7–11.4)	< 0.001
Medications during hospital stay				
Antivirals	3.7 (2.9–4.7)	< 0.001	1.6 (0.9–2.8)	0.111
Steroid therapy	6.4 (5.4–7.8)	< 0.001	1.3 (0.9–2.1)	0.179
Hydroxychloroquine	0.7 (0.6–0.8)	< 0.001	0.6 (0.4–1.1)	0.051
Intravenous immunoglobulin	8.6 (4.3–17.5)	< 0.001	1.6 (0.4–6.4)	0.480
Convalescent plasma	23.2 (9.4–57.3)	< 0.001	3.7 (0.9–15.8)	0.074

OR = odds ratio; qSOFA = quick Sequential Organ Failure Assessment; Ref = reference category; SpO_2_ = oxygen saturation.

* Adjusted for age, sex, comorbidities, presenting symptoms, SpO_2_, and qSOFA score at admission, admission as a Covid patient, intensive care unit admission, invasive mechanical ventilation, and associated complications during hospital stay and hospital where cases were recruited.

## DISCUSSION

In this hospital-based case-control study of predictors of mortality due to COVID-19, we found that older age, preexisting diabetes mellitus, pulmonary tuberculosis or malignancy, difficulty in breathing at admission, and low SpO_2_ and high qSOFA score at time of admission were associated with higher odds of death. Patients with COVID-19 who were 75 years or older were 11 times more likely to die than those younger than 30 years. To the best of our knowledge, this is one of the first multicentric studies to estimate predictors of COVID-19 death in India with a robust sample size.

We found that increasing age was associated with increased odds of mortality due to COVID-19. This is consistent with evidence from elsewhere. A multicentric cohort study among adults 18 years or older in 10 countries of Africa found that every increasing year increased the odds of death due to COVID-19 by 3%.^14^ A similar association was reported in a meta-analysis as well in studies conducted in other countries.^6^^,^^7^^,^^9^^,^^14^^–^^18^ We did not find any association between the sex of the patient and the odds of mortality due to COVID-19. Available evidence from elsewhere in this regard is conflicting. Although two meta-analyses and some other studies found that males were more likely to die as a result of COVID-19 in-hospital,^6^^,^^7^^,^^17^^,^^19^ other studies did not find any association.^9^^,^^14^^,^^20^ Another study found a significant association between male sex and in-hospital mortality due to COVID-19 only in those older than 65 years.^18^

We found that poor clinical status at the time of hospital admission was associated with increased odds of mortality. The qSOFA score, which combines low systolic blood pressure, altered mental status, and raised respiratory rate, was a strong predictor of mortality due to COVID-19 in our study. We found that those with a qSOFA score of 2 and above were five times more likely to die as a result of COVID-19, which was consistent with other studies.^14^^,^^20^ An SpO_2_ value ≤ 94% at the time of admission was also associated with higher odds of mortality; similar results were found in other studies as well.^9^^,^^17^^,^^21^ We found a strong association between difficulty breathing at the time of admission and mortality, although the presence of a cough and fever was not significantly associated. Most of the studies conducted across different settings found a positive association between breathlessness at the time of admission and mortality^8^^,^^16^^,^^17^^,^^21^; however, evidence regarding cough is conflicting. While many studies have found cough to be a protective factor in COVID-19 mortality,^8^^,^^17^ we could not find any such association. Most of the studies did not find any association between fever and mortality due COVID-19.^7^^,^^8^^,^^16^

Among the preexisting morbidities that we examined, diabetes mellitus, malignancy, and pulmonary tuberculosis were found to be independent predictors of mortality due to COVID-19. Studies have found an association between multi-morbidity and deaths due to COVID-19.^22^ Although patients with diabetes mellitus were almost twice as likely as those without diabetes mellitus to die as a result of COVID-19, those with any malignancy were three times more likely to die. Most of the available evidence suggests that diabetes mellitus is one of the most important predictors of in-hospital COVID-19 mortality,^6^^,^^8^^,^^12^^,^^14^^,^^23^ whereas evidence regarding malignancy and other morbidities is conflicting. Although some studies found an association between malignancy and COVID-19 mortality,^15^^,^^17^^,^^23^ others did not find any association.^9^^,^^14^ One study found an association only among males.^18^ We did not find any association between COVID-19 mortality and ischemic heart disease, hypertension, chronic respiratory disease, or stroke.

We found that use of hydroxychloroquine was not associated with reduced odds of mortality due to COVID-19. Although some observational studies have reported protection with use of hydroxychloroquine,^9^ most trials did not find any effect of hydroxychloroquine on reducing 28-day mortality.^10^^,^^11^^,^^24^ We did not find any association between steroid use and mortality due to COVID-19. A large trial found that dexamethasone reduced 28-day mortality from COVID-19 among those receiving oxygen support or on mechanical ventilation, but not among those not receiving any respiratory support.^25^ However, even after we restricted our analysis to only those patients receiving respiratory support, steroid therapy was not associated with reduced odds of mortality. We were unable to collect information on type or duration of steroid therapy; however, methylprednisolone was the most common steroid used. We were also unable to collect information on duration of use of other medications. However, we found that convalescent plasma therapy did not provide any survival benefit. Most trials did not find any association between therapy with convalescent plasma and protection from mortality due to COVID-19.^26^^,^^27^

A similar study conducted in Tamil Nadu (South India) found a higher mortality rate among patients aged 40–70 years, with the highest rate among diabetic patients with elevated urea levels.^28^ The aORs of significant factors in the multivariable logistic regression were SpO_2_ < 95%: 2; age ≥ 50 years: 2.52; pulse rate ≥ 100/minute: 2.02; and coexisting diabetes mellitus: 1.73, with hypertension and gender not retaining their significance. These results are similar to those of the current study in relation to the variables SpO_2_ (95/94%), age, and preexisting diabetes mellitus.

### Strengths and limitations of the study.

This study was conducted in multiple hospital settings with a robust sample size. However, there are some limitations. As is true for any study based on retrospective extraction of patient case records, completeness was a major limitation. The study did not capture risk factors such as day of illness at admission, severity of illness (moderate/severe), or laboratory parameters, as well as details of treatment such as dose and duration. However, we used indicators for severity both at the time of admission (e.g., qSOFA score) and during the period of hospitalization (e.g., need for ventilatory support, ICU admission, and associated complications in the hospital) to adjust our analyses. We did not impute any data on signs and symptoms, but preexisting morbidities or treatment modalities during hospitalization were considered positive only when they were explicitly mentioned in the case record. The ratio of cases to controls in the study institutions was variable. The emergence of newer data during the study period led to a change in standard practice at the institutions in the study period, which may have affected the comparisons.

## CONCLUSION

In this hospital-based case-control study, we found that older age, preexisting diabetes mellitus, pulmonary tuberculosis or malignancy, difficulty in breathing, low SpO_2_ and general condition as determined by qSOFA score at the time of admission, and admission to the ICU were significant predictors of mortality among hospitalized patients with COVID-19. Prioritizing patients at increased risk of death and rationalizing therapy will help reduce mortality due to COVID-19 during the ongoing pandemic.
